# Long-Term Exposure to Cigarette Smoke Extract Induces Hypomethylation at the RUNX3 and IGF2-H19 Loci in Immortalized Human Urothelial Cells

**DOI:** 10.1371/journal.pone.0065513

**Published:** 2013-05-28

**Authors:** Li-Mei Chen, Jennifer C. Nergard, Liqiang Ni, Charles J. Rosser, Karl X. Chai

**Affiliations:** 1 Burnett School of Biomedical Sciences, College of Medicine, University of Central Florida, Orlando, Florida, United States of America; 2 Department of Statistics, College of Sciences, University of Central Florida, Orlando, Florida, United States of America; 3 Cancer Research Institute, M.D. Anderson Cancer Center Orlando, Orlando, Florida, United States of America; 4 Section of Urologic Oncology, M.D. Anderson Cancer Center Orlando, Orlando, Florida, United States of America; University of Bristol, United Kingdom

## Abstract

Cigarette smoking is the single most important epidemiological risk factor for bladder cancer but it is not known whether exposure of urothelial cells to the systemic soluble contents of cigarette smoke is directly causative to bladder cancer and the associated epigenetic changes such as tumor suppressor gene hypermethylation. We undertook this study to investigate if long-term treatment of human urothelial cells with cigarette smoke extract (CSE) results in tumor suppressor gene hypermethylation, a phenotype that was previously associated with long-term constant CSE treatment of airway epithelial cells. We chronically treated an immortalized human urothelial cell line UROtsa with CSE using a cyclic daily regimen but the cells were cultured in CSE-free medium between daily treatments. Bisulfite sequencing and real-time PCR array-based methylation profiling were employed to evaluate methylation changes at tumor suppressor gene loci in the chronically CSE-treated cells versus the passage-matched untreated control cells. The RUNX3 tumor suppressor gene promoter was hypomethylated with a significant increase in proportion of the completely unmethylated haplotype after the long-term CSE treatment; whereas RUNX3 promoter hypermethylation was previously reported for bladder cancers of smokers. Hypomethylation induced by the long-term CSE treatment was also observed for the IGF2-H19 locus. The methylation status at the PRSS8/prostasin and 16 additional loci however, was unaffected by the chronic CSE treatment. Transient CSE treatment over 1 daily regimen resulted in transcriptional down-regulation of RUNX3 and H19, but only the H19 transcription was down-regulated in the chronically CSE-treated urothelial cells. Transcription of a key enzyme in one-carbon metabolism, dihydrofolate reductase (DHFR) was greatly reduced by the long-term CSE treatment, potentially serving as a mechanism for the hypomethylation phenotype via a reduced supply of methyl donor. In conclusion, chronic cyclic CSE treatment of urothelial cells induced hypomethylation rather than hypermethylation at specific loci.

## Introduction

Cigarette smoking is the single most important epidemiological risk factor for bladder cancer as smokers have a four-fold higher risk versus nonsmokers; while about half of bladder cancers among men and women may be associated with or attributed to cigarette smoking [1]. The mainstream cigarette smoke or the cigarette smoke condensate/extract (CSC/CSE) has been shown to cause all sorts of genomic DNA damages in experimental animals as well as in humans, and is regarded as a systemic human mutagen with a direct role in causing eight different cancers including that of the urinary bladder [2]. In recent years, the role of epigenetic/epigenomic modifications in carcinogenesis, at all stages including initiation, promotion, and progression has been recognized. Bladder cancer was the model with which Peter Jones and colleagues expanded the “Knudson two-hit hypothesis” to include the mechanism of tumor suppressor silencing by epigenetic modifications in the consideration in addition to the mechanism of DNA damages [3]. The molecular pathways that lead to the development of papillary, low-grade, non-invasive urothelial carcinomas versus those to the development of the invasive type are believed to be distinct and mutually exclusive. Aberrant DNA methylation correlates with increased bladder tumor invasion and progression and is implicated for a causative role [3]. In the non-muscle invasive urothelial carcinomas, DNA methylation at six of twenty cancer-associated genes examined in a 105-patient cohort was shown to associate with recurrence, while methylation at one of the genes predicts prolonged disease-free interval [4].

In bladder cancers, promoter DNA hypermethylation in tumor suppressor genes has been associated with cigarette smoking [5]. Promoter methylation in the p16-INK4A gene in bladder cancers was associated with cigarette smoking, with a greater than doubled risk for smokers versus nonsmokers, and with a greater risk in patients with more recent history of smoking [6]. DNA methylation at the RUNX3 (runt-related transcription factor 3) tumor suppressor gene naturally increases with age and occurs significantly earlier in bladder cancers of smokers than nonsmokers [7]. The associated effects of cigarette smoking on bladder cancer development appear direct, early, and more aggressive than the effects of other factors.

While the epidemiological evidence is strong for a causative role of cigarette smoking in bladder cancer development and in epigenetic changes in bladder cancer specimens, there is no direct proof that exposure of urothelial cells to cigarette smoke causes these changes. A recent study reported that long-term exposure of airway epithelial cells to cigarette smoke condensate/extract resulted in cellular transformation marked by increased soft-agar clonogenicity and induced hypomethylation of repetitive DNA but hypermethylation of tumor suppressor genes; epigenetic changes that are consistent with pulmonary carcinogenesis [8]. In our study presented here, we exposed urothelial cells to cigarette smoke extract (CSE) and evaluated CpG methylation changes in two marker genes, RUNX3 and PRSS8/prostasin in the CSE-treated cells versus the untreated control cells. The PRSS8/prostasin serine protease is down-regulated in bladder cancer and its loss in bladder cancer cell lines correlates with promoter DNA hypermethylation [9]. We then performed a methylation-specific PCR array analysis of 24 human tumor suppressor genes on the CSE-treated cells and the untreated control cells. DNA hypomethylation in the RUNX3 promoter, the IGF2 (insulin-like growth factor II) gene DMR (differentially methylated region), and the H19 CBS6 (CTCF-binding site 6) was observed in association with long-term CSE treatment of urothelial cells; along with down-regulation of dihydrofolate reductase (DHFR) expression.

## Materials and Methods

### Cell culture

The immortalized human urothelial cell line UROtsa was cultured as described previously [9].

### CSE preparation

The preparation of CSE was described previously [10]. Briefly, research-reference cigarettes (1R3F, 1.16 mg nicotine and 15 mg tar) were obtained from the University of Kentucky, Tobacco Research and Development Center, and kept in a refrigerated dry environment, but equilibrated to room temperature before use. CSE was routinely prepared by bubbling one unfiltered cigarette through 25 ml of serum-free DMEM/F12 medium [11]. The CSE stock medium was then filtered through a 0.22-µm filter to remove particles and microbes. Large stocks of CSE were prepared as described above and designated as 100%, and stored at –80°C in small aliquots for use within two weeks. Each aliquot was used only once after thawing. The 100% CSE prepared by the procedures described above was estimated to represent approximately 1/4 of the average smoker’s plasma concentration of soluble cigarette smoke contents [12]. The CSE was diluted to the desired concentrations in culture medium when used.

### CSE treatment of cells

UROtsa cells were seeded in a T25 flask and grown to 60-70% confluence prior to treatment. In one cycle of treatment, the cells were treated for 15 minutes with 50% CSE diluted in serum-free DMEM/F12 medium, and then cultured for 1 hour in CSE-free fresh growth medium (DMEM/5%FBS/1 mM Glucose). The treatment was repeated for 3 cycles per day and between daily treatments the cells were cultured in the CSE-free growth medium. The daily CSE treatments were performed for 4 days consecutively before the cells were passaged (at 1:5) into a new T25 flask for continuation of the long-term treatment.

### Bisulfite sequencing

High-molecular-weight genomic DNA was extracted from the UROtsa cell samples as described previously [13]. Bisulfite (BS) treatment of the genomic DNA was performed by using the EpiTect Bisulfite Kit (QIAGEN, Valencia, CA) and the supplier’s protocol. For each sample, 2 μg of starting genomic DNA were used for the BS treatment. For amplification of the RUNX3 and PRSS8 gene fragments for sequencing analysis, 10% of each BS-treated DNA sample was used, with the primers listed below for each target gene.

PRSS8: forward: GGGGGTATAATTGGTTTGAGATAAT

reverse: CCTCCCAAAAATAACTACACCTACC

The primers for RUNX3 promoter fragment amplification off the BS-DNA were described previously [7].

The top strand of the BS-treated DNA was amplified for each target gene, using the GoTaq Hot Start Polymerase (Promega, Madison, WI). Amplification of the IGF2 DMR and H19 CBS6 was performed as described previously [14]. The PCR amplicons were purified using the QIAEX II Gel Extraction Kit (QIAGEN) after agarose gel electrophoresis, and cloned into the pGEM-T Easy plasmid (Promega). For each sample, 60 independent clones were selected for sequencing, performed by Eton Bioscience (Research Triangle Park, NC).

### Methylation PCR array analysis

A Methyl-Profiler DNA Methylation PCR Array for Human Tumor Suppressor Genes, Signature Panel MeAH-551A (QIAGEN) was used to analyze the methylation changes of 24 genes using the supplier’s protocol.

### Quantitative real-time PCR analysis

Total cellular RNA isolation, reverse transcription and real-time PCR were performed as described previously [10]. The primers used in the quantitative evaluation of gene expression were:

H19: forward: CGTCCCTTCTGAATTTAATTTG

reverse: ACACTCGTACTGAGACTC

DNMT1: forward: GAACCAACACCCAAACAG

reverse: TTCTCGTCTCCATCTTCG

The primers used for RUNX3, IL-6, DHFR, and 18S rRNA were described previously [15], [16], [17], [18]. The relative mRNA expression of each target gene was calculated as the copy number per 1/100 18S rRNA copy.

### Western blot analysis

Evaluation of protein expression changes by western blot analysis was performed as described previously [10]. The antibodies used were DHFR (used at 1∶1,000 dilution) and GAPDH (glyceraldehyde 3-phosphate dehydrogenase, used at 1∶5,000 dilution) (Santa Cruz Biotechnology). The detected signals were quantified by densitometric analysis using an Alpha Imager 2200 (Alpha Innotech Corporation) with the AlphaEaseFC Software (Version 3.2.1). The relative protein expression of DHFR in each sample was calculated using the GAPDH protein level as the normalization standard.

### Statistical analysis

Student’s t-test was used to evaluate comparisons of two means, whereas Z-test was used to evaluate comparisons of two percentages. Differences were considered statistically significant when *p*<0.05.

## Results

### The RUNX3 promoter is hypomethylated in urothelial cells after long-term CSE exposure

For the chronic treatment regimens we needed to determine the concentration of CSE for use so as to produce the maximum effect on the marker gene expression during the treatment intervals but with the least toxicity on the cells. We used RUNX3 mRNA expression down-regulation as an indicator for the effects of CSE treatment at various concentrations, and cell growth as a measure of toxicity, as described in a previous study [10]. We tested 10%, 20%, 50% and 100% CSE by these criteria, and 50% CSE was found to down-regulate RUNX3 mRNA expression by the greatest margin without causing major impediments on cell proliferation (data not shown).

**Figure 1:**
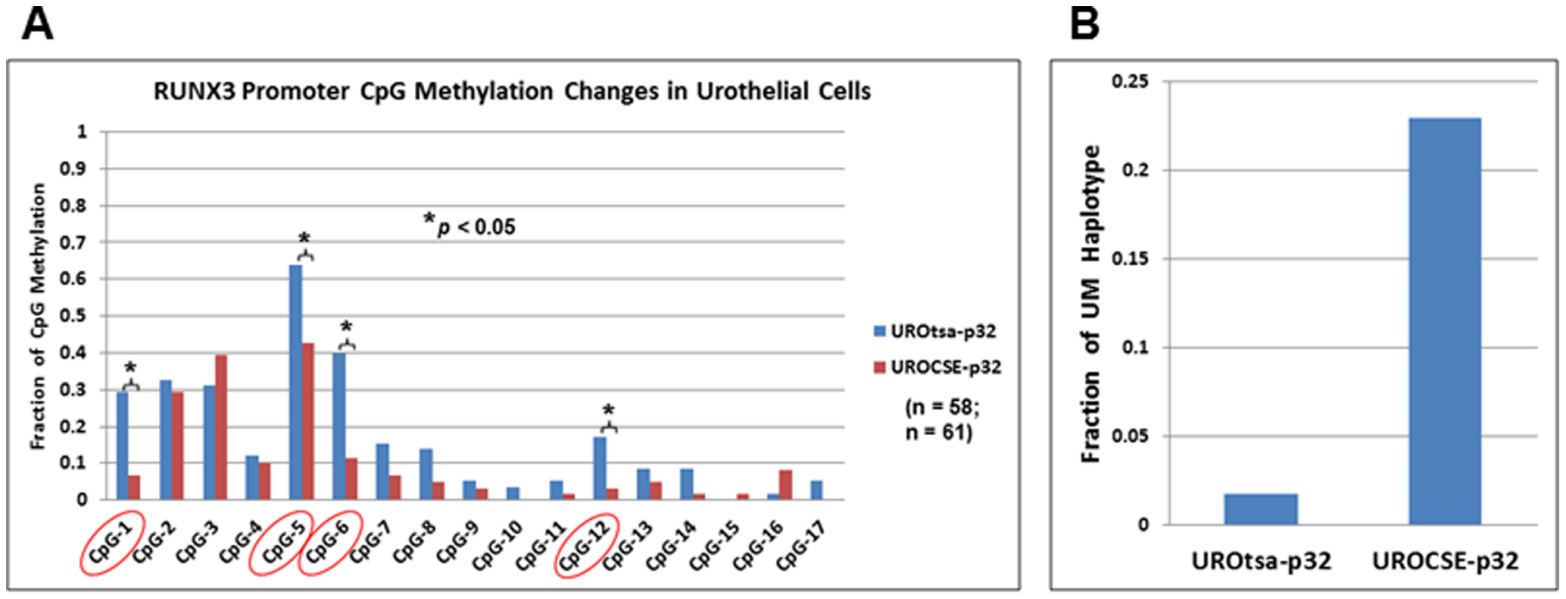
Epigenetic changes at the RUNX3 locus in UROtsa cells chronically treated with CSE. A. Percentage of methylation at 17 CpG sites in the RUNX3 promoter determined by bisulfite sequencing of genomic DNA extracted from UROtsa cells chronically treated with CSE, UROCSE-p32 (32^nd^ passage) and from untreated control cells, UROtsa-p32 (32^nd^ passage). Percentage of methylation was calculated by the number of methylated cytosines at each CpG site observed in the total number of independent clones sequenced for each BS DNA amplicon, for UROtsa-p32, n = 58; and for UROCSE-p32, n = 61. The encircled sites presented hypomethylation in the UROCSE-p32 cells (*p*<0.05). B. Fractions of completely unmethylated (UM) haplotype in the UROtsa-p32 and the UROCSE-p32 cells.

The RUNX3 BS DNA amplicon subjected to sequencing analysis contains 17 CpG sites in the promoter region of this gene, including 2 sites (designated as CpG-1 and CpG-2 in this amplicon) previously shown to be hypermethylated in bladder cancer specimens from smokers [7]. We examined the RUNX3 promoter in CSE-treated UROtsa cells of the 32^nd^ passage (designated as UROCSE-p32), and in passage-matched control cells (UROtsa-p32) cultured in CSE-free growth medium. The data were first analyzed for each individual CpG site by calculating the percentage of independent clones presenting the methylated cytosine. Of the 17 CpG sites examined, 4 sites, CpG-1, -5, -6, and -12, were found to be hypomethylated in the UROCSE-p32 cells versus the UROtsa-p32 cells (*p*<0.05; ***Figure 1A***). Methylation changes at all other CpG sites did not reach statistical significance (*p*>0.05).

We then analyzed each clone to determine if there were epigenetic haplotype changes associated with the CSE treatment. As shown in ***Figure 1B***, approximately 23% (14/61) of the RUNX3 BS DNA clones of the UROCSE-p32 cells were completely unmethylated (UM) at all 17 CpG sites, whereas the UROtsa-p32 cells had less than 2% (1/58) completely unmethylated clones.

### CpG methylation in the PRSS8 promoter is unaffected in urothelial cells after long-term CSE exposure

**Figure 2:**
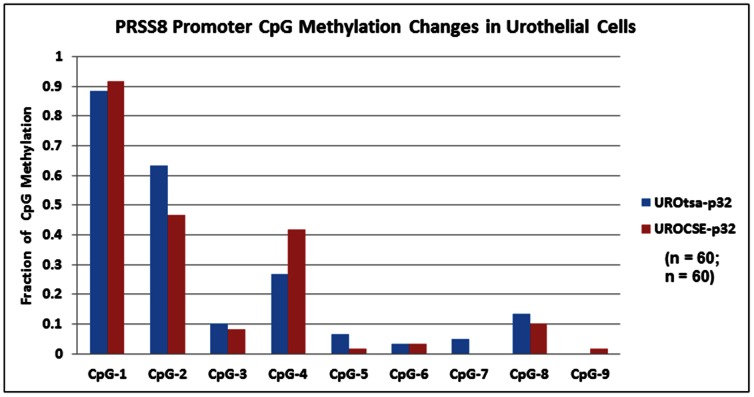
Epigenetic changes at the PRSS8 locus in UROtsa cells chronically treated with CSE. Percentage of methylation at 9 CpG sites in the PRSS8 promoter determined by bisulfite sequencing of genomic DNA extracted from UROtsa cells chronically treated with CSE, UROCSE-p32 (32^nd^ passage) and from untreated cells, UROtsa-p32 (32^nd^ passage). Percentage of methylation was calculated by the number of methylated cytosines at each CpG site observed in the total number of independent clones sequenced for each BS DNA amplicon, n = 60 for both UROtsa-p32 and UROCSE-p32.

The PRSS8 BS DNA amplicon subjected to sequencing analysis contains 9 CpG sites in the promoter region of this gene. We have shown previously that hypermethylation at some of these sites (CpG-3 and CpG-5) is causative to PRSS8 expression down-regulation in human bladder, breast, and prostate cancer cell lines [9], [13], [19]. Between the UROCSE-p32 cells and the UROtsa-p32 cells, no methylation changes at any of the CpG sites reached statistical significance (*p*>0.05, ***Figure 2***). One completely unmethylated (UM) amplicon was among the independent clones sequenced for the UROCSE-p32 cells but none was seen for the UROtsa-p32 cells.

### Methylation PCR array analysis reveals hypomethylation at the IGF2 gene locus

The Methyl-Profiler array with 24 human tumor suppressor genes was applied to analyze the genomic DNA of the UROCSE-p32 and the UROtsa-p32 cells. Of the 21 genes that had successful amplification according to the supplier’s quality control standards, 17 genes had less than 1% of change in either Hyper- (HM), Intermediately- (IM), or Un-methylated (UM) DNA between the CSE-treated cells and the untreated control cells (***Figure S1***). Three (3) other genes had 2–11% of changes in their methylation status between the two cell types. The methylation status change of IGF2 was the greatest. The Hyper-methylated (HM) IGF2 DNA was at ∼61% in the untreated control UROtsa-p32 cells, but at 50% in the CSE-treated UROCSE-p32 cells; with a corresponding change of Un-methylated (UM) DNA from ∼39% to 50%.

**Figure 3:**
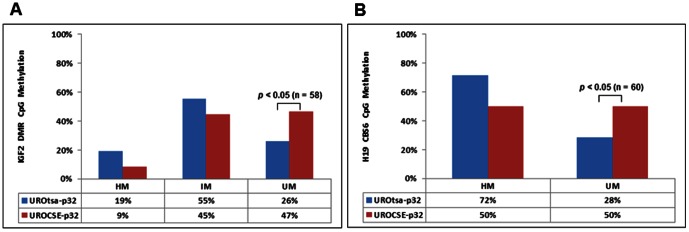
Epigenetic changes at the IGF2-H19 locus in UROtsa cells chronically treated with CSE. A. Percentage of methylation at 3 CpG sites in the IGF2 DMR determined by bisulfite sequencing of genomic DNA extracted from UROtsa cells chronically treated with CSE, UROCSE-p32 (32^nd^ passage) and from untreated cells, UROtsa-p32 (32^nd^ passage). HM: hypermethylated with 3 of 3 CpG sites presenting methylation; IM: intermediately methylated with 2 or 1 of 3 CpG sites presenting methylation; UM: unmethylated with 0 of 3 CpG sites presenting methylation; n = 58 for both UROtsa-p32 and UROCSE-p32. The increase of UM DNA at this locus in the UROCSE-p32 cells was statistically significant (*p*<0.05), as indicated in the figure. B. Percentage of methylation at 23 CpG sites in the H19 CBS6 determined by bisulfite sequencing of genomic DNA extracted from UROtsa cells chronically treated with CSE, UROCSE-p32 (32^nd^ passage) and from untreated cells, UROtsa-p32 (32^nd^ passage). HM: hypermethylated with >70% of the CpG sites presenting methylation; UM: unmethylated with 0 of 23 CpG sites presenting methylation; n = 60 for both UROtsa-p32 and UROCSE-p32. One clone presenting 8 methylated CpG sites of the 23 was observed for the UROCSE-p32 cells and was counted as UM. The increase of UM DNA at this locus in the UROCSE-p32 cells was statistically significant (*p*<0.05), as indicated in the figure.

As IGF2, along with the neighboring H19 gene, are genomically imprinted in humans, we interrogated in further detail the methylation status changes in the CSE-treated and the untreated control UROtsa cells by amplifying the IGF2 DMR and the H19 CBS6 from the BS DNA for sequencing analysis. The IGF2 DMR BS DNA amplicon contains 3 CpG sites, and we grouped the clones by their methylation status as Hyper-methylated (HM, 3/3), Intermediately-methylated (IM, 1/3, or 2/3), and Un-methylated (UM, 0/3). As shown in ***Figure 3A***, long-term CSE treatment had a hypomethylation effect on the IGF2 DMR, increasing the UM DNA from ∼26% (15/58) in the UROtsa-p32 cells to ∼47% (27/58) in the UROCSE-p32 cells (*p*<0.05). The H19 CBS6 BS DNA amplicon contains 23 CpG sites. We dichotomized the methylation status as Hyper-methylated (HM) versus Un-methylated (UM) because all clones for both the UROCSE-p32 and the UROtsa-p32 cells were either >70% methylated (HM) or completely unmethylated (UM) at the 23 sites, with one exception among the independent clones sequenced for the UROCSE-p32 cells. This clone was ∼35% (8/23) methylated and was counted as UM. As shown in ***Figure 3B***, H19 CBS6 is hypomethylated in the UROCSE-p32 cells when compared to the UROtas-p32 cells (*p*<0.05).

Two loci, RARB (retinoic acid receptor beta) and RASSF1 (Ras association domain family 1), showed hypermethylation in airway epithelial cells following chronic constant CSE treatment [8], but are minimally hypermethylated in the chronically but cyclically CSE-treated urothelial cells (***Figure S1***). The RUNX3 locus however, was also reported to have a <1% methylation change by the array analysis (***Figure S1***).

### Hypomethylated loci show corresponding repression of expression in the chronically CSE-treated cells

**Figure 4:**
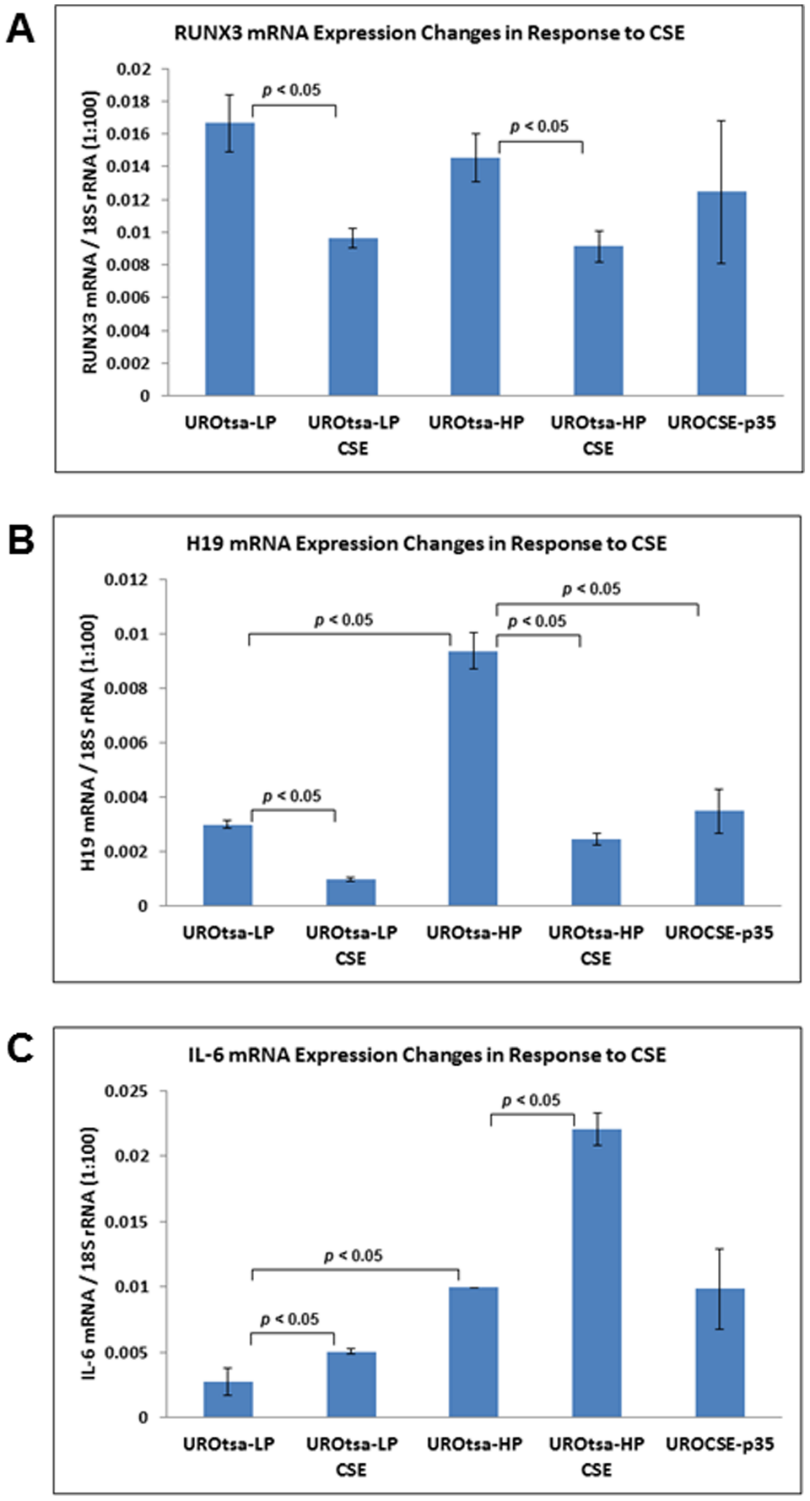
Expression changes of genes presenting CSE-induced hypomethylation in the CSE-treated UROtsa cells. A. RUNX3 mRNA expression in UROtsa cells transiently or chronically treated with CSE. Data represent RUNX3 mRNA copy numbers relative to 1/100 of the 18S rRNA copy number, determined as the mean of three independent experiments with standard errors shown by the error bars. UROtsa-LP: UROtsa cells cultured in CSE-free medium, 8^th^ passage; UROtsa-LP CSE: UROtsa-LP cells treated for 1 day with CSE. UROtsa-HP: UROtsa cells cultured in CSE-free medium, 34^th^ passage; UROtsa-HP CSE: UROtsa-HP cells treated for 1 day with CSE. UROCSE-p35: UROtsa cells chronically treated with CSE, 35^th^ passage. Statistically significant differences between the datum columns are as indicated in the figure (n = 3, *p*<0.05). B. H19 mRNA expression in UROtsa cells transiently or chronically treated with CSE. Statistically significant differences between the datum columns are as indicated in the figure (n = 3, *p*<0.05). C. IL-6 mRNA expression in UROtsa cells transiently or chronically treated with CSE. Statistically significant differences between the datum columns are as indicated in the figure (n = 3, *p*<0.05).

We investigated if the DNA methylation changes that we observed in the chronically CSE-treated UROtsa cells are associated with transcriptional changes of the genes evaluated. The RUNX3 mRNA expression was not affected by the chronic CSE treatment, when evaluated between UROCSE-p35 and UROtsa-p34 (shown as UROtsa-HP in ***Figure 4A***). We also evaluated RUNX3 mRNA expression in response to a transient CSE treatment for 3 treatment cycles but 1 day only, using low-passage UROtsa cells (p8, shown as UROtsa-LP) as well as high-passage UROtsa cells (p34, UROtsa-HP). In the UROtsa-LP and the UROtsa-HP cells, transient CSE treatment resulted in reduced RUNX3 mRNA expression, by ∼42% and ∼37%, respectively (*p*<0.05) (***Figure 4A***). Passaging had no effect on RUNX3 mRNA expression (comparing UROtsa-LP and UROtsa-HP, *p*>0.05). The PRSS8 mRNA expression was not affected by the chronic CSE treatment, whereas the IGF2 mRNA expression level fell below the sensitivity limit of the assay (data not shown). The H19 mRNA expression was lower in the UROCSE-p35 cells as compared to the UROtsa-p34 (HP) cells by ∼63% (*p*<0.05, ***Figure 4B***). Transient CSE treatment resulted in reduced H19 mRNA expression in the UROtsa-LP and UROtsa-HP cells, by ∼68% and ∼74%, respectively (*p*<0.05, ***Figure 4B***). Passaging however, had an apparently up-regulatory effect on H19 mRNA expression, resulting in a ∼3-fold higher expression in the UROtsa-HP cells versus the UROtsa-LP cells (*p*<0.05, ***Figure 4B***).

We evaluated the interleukin-6 (IL-6) mRNA expression in the five cell types described above to validate the CSE treatments, as CSE has been shown to up-regulate IL-6 expression in cultured cells [20]. In both the UROtsa-LP and the UROtsa-HP cells, the transient CSE treatment resulted in IL-6 mRNA up-regulation, to ∼1.8 and ∼2.2 fold, respectively (*p*<0.05, ***Figure 4C***). But there was no difference of IL-6 mRNA level between the UROCSE-p35 and the UROtsa-p34 (HP) cells (*p*>0.05, ***Figure 4C***). IL-6 mRNA expression was ∼3.6 fold higher in the UROtsa-HP cells than in the UROtsa-LP cells (*p*<0.05, ***Figure 4C***).

### DHFR expression was reduced in association with CSE treatment

**Figure 5:**
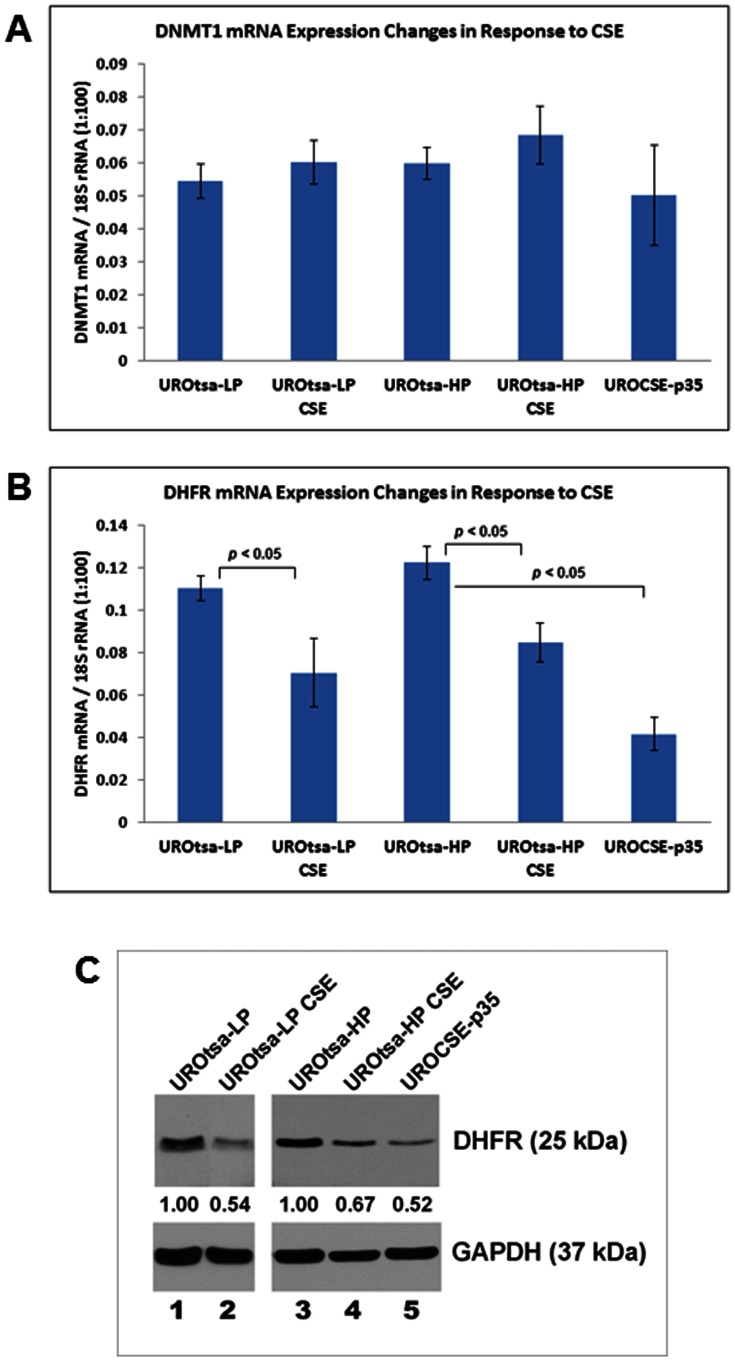
Changes of DNMT1 and DHFR expression in the CSE-treated UROtsa cells. A. DNMT1 mRNA expression in UROtsa cells transiently or chronically treated with CSE. Data represent DNMT1 mRNA copy numbers relative to 1/100 of the 18S rRNA copy number, determined as the mean of three independent experiments with standard errors shown by the error bars. UROtsa-LP: UROtsa cells cultured in CSE-free medium, 8^th^ passage; UROtsa-LP CSE: UROtsa-LP cells treated for 1 day with CSE. UROtsa-HP: UROtsa cells cultured in CSE-free medium, 34^th^ passage; UROtsa-HP CSE: UROtsa-HP cells treated for 1 day with CSE. UROCSE-p35: UROtsa cells chronically treated with CSE, 35^th^ passage. B. DHFR mRNA expression in UROtsa cells transiently or chronically treated with CSE. Statistically significant differences between the datum columns are as indicated in the figure (n = 3, *p*<0.05). C. DHFR protein expression in UROtsa cells transiently or chronically treated with CSE. The images presented here represent the results from three independent experiments. For samples in Lanes 1 and 2, 40 µg of total protein were loaded in each lane; for samples in Lanes 3–5, 15 µg of total protein were loaded in each lane. After normalizing the DHFR signal intensities to the corresponding GAPDH signal intensities, the relative DHFR signal intensity in the untreated cells was assigned as 1.00 and the relative DHFR signal intensities of the CSE-treated cells are as indicated in the figure.

To explore the potential molecular mechanisms underlying the hypomethylation phenotypes observed at the RUNX3 and the IGF2-H19 loci in the chronically CSE-treated urothelial cells, we evaluated the mRNA expression of DNA methyltransferases (DNMT1, DNMT3A, and DNMT3B), and of the key players in folate metabolism (dihydrofolate reductase, DHFR; folate receptor, FOLR1; and folate transporter, RFC1). The DNMT1 mRNA expression was unaffected by either the chronic or the transient CSE treatment (*p*>0.05, ***Figure 5A***), whereas the DNMT3A and DNMT3B mRNA levels fell below the sensitivity limit of the assay (data not shown).

The DHFR mRNA expression was lower in the UROCSE-p35 cells as compared to the UROtsa-p34 (HP) cells, by ∼66% (*p*<0.05, ***Figure 5B***). Transient CSE treatment resulted in reduced DHFR mRNA expression as well, in both the UROtsa-LP and the UROtsa-HP cells, by ∼36% and ∼31%, respectively (*p*<0.05, ***Figure 5B***). Passaging had no effect on DHFR mRNA expression (comparing UROtsa-LP and UROtsa-HP, *p*>0.05). The FOLR1 and RFC1 mRNA levels fell below the sensitivity limit of the assay (data not shown). The down-regulation of DHFR transcription by the CSE treatments was accompanied with a corresponding down-regulation of DHFR protein expression, as shown in ***Figure 5C***.

## Discussion

We undertook this study to determine if direct exposure of cultured urothelial cells to CSE over the long term, mimicking the exposure of urothelial cells to the systemic soluble contents of cigarette smoke in smokers, would have epigenetic modification effects on the urothelial cells. The study design was to monitor the epigenetic changes in two tumor suppressor genes, RUNX3 and PRSS8, and follow up with a screening for epigenetic changes in more tumor suppressor genes if CSE-induced changes were to be observed in the RUNX3 and PRSS8 marker genes. We had fully expected that both of these marker genes would present a hypermethylation phenotype in the chronically CSE-treated urothelial cells as RUNX3 hypermethylation in bladder cancer is associated with smoking [7] and PRSS8 hypermethylation correlates with bladder cancer progression [9]. The RUNX3 promoter however, showed hypomethylation at CpG-1 in the urothelial cells chronically exposed to CSE, whereas this same site was found to be hypermethylated in bladder cancer specimens of smokers.

The time course of CSE treatment of the urothelial cells in our study is comparable to the time course of CSE treatment of the airway epithelial cells in the study by Liu et al. [8], in which tumorigenic transformation and hypermethylation of tumor suppressor genes were reported for the chronically CSE-treated airway epithelial cells. At between the 32^nd^ and the 35^th^ passages, the UROtsa cells chronically treated with CSE by our regimen (3 cycles a day with 15 minutes of exposure per cycle using 50% CSE) were not transformed as no colonies could form in soft agar anchorage-independent growth assays (data not shown). The UROtsa cell line is a transformation-competent cell line, capable of forming colonies in soft agar upon tumorigenic transformation, as shown previously by others [21]. With these observations, we tentatively conclude that chronic CSE treatment alone is not sufficient to transform cultured urothelial cells.

There is however, a major technical difference in our urothelial cell chronic CSE treatment regimen versus that used in the study by Liu et al. to treat the airway epithelial cells, which were constantly exposed to CSE for the 5 months of treatment with daily changes of CSE-supplemented medium [8]. We reason that our cyclic treatment regimen is more appropriate for evaluating the effects of systemic soluble cigarette smoke contents on tissues that are not directly exposed to the cigarette smoke. Constant CSE exposure would effectively simulate non-stop smoking whereas the cyclic CSE exposure would more closely mimic the behavioral patterns of smokers over a 24-hour daily period. It is therefore, not surprising that loci with >100% increase in methylation in the chronically CSE-treated airway epithelial cells under constant exposure, e.g., RARB and RASSF1 [8], were only minimally affected in the urothelial cells under cyclic exposure (***Figure S1***). We were unable to perform a direct comparison of methylation changes in UROtsa cells treated cyclically versus those treated constantly over the long term because the 50% CSE concentration used in the cyclic regimens proved to be much too cytotoxic when present in the cultures constantly and would not have allowed successive passaging of the treated cells. The wisdom of using the cyclic or the constant regimen for chronic CSE treatment of cultured cells to study the effects of CSE on these cells may be open for debate and the cell type differences between the airway epithelial and the urothelial cells notwithstanding, the research field should now carefully consider the differences in the phenotypes induced by the two different treatment regimens.

It must also be emphasized that cigarette smoke contents may undergo metabolic activation in the body and thus the in vivo effects on the cells would be brought forth by the original components as well as the metabolites, whereas treating cultured cells in vitro with CSE may or may not produce the effects of the metabolites depending on the cell types. Despite this limitation, adding CSE to the culture medium to test its immediate effects on cultured cells in vitro was used by many published studies that provided meaningful molecular and cellular mechanistic insights on the impact of CSE to the cells in various physiological contexts [2], [8], [10], [11].

There is a possibility that the Methyl-Profiler DNA Methylation PCR Array that we employed for evaluation of methylation changes at select tumor suppressor gene loci could report false negative results. The RUNX3 locus was among the 24 genes in the Methyl-Profiler array, but it was determined to have a rather minimal hypomethylation change in the UROCSE-p32 cells as compared to the UROtsa-p32 cells when the array analyses were performed (***Figure S1***). In addition, the methylation status of the RUNX3 gene reported for the untreated UROtsa cells was ∼99% hypermethylated, in apparent contradiction to the previous reports by others showing an unmethylated to intermediately methylated status at this locus in normal urothelial cells [7], [22]. The study by Kim et al. [22] examined the CpG sites in the immediately upstream region of the RUNX3 gene, from nucleotide -211 to nucleotide -61; whereas the region inspected in the study by Wolff et al. [7] was from nucleotide -820 to nucleotide -601, further upstream. Kim et al. [22] used direct sequencing to determine the methylation status of all 25 CpG sites in the (−211 to −61) region; whereas Wolff et al. [7] looked at the average of 2 of 17 CpG sites in the (−820 to −601) region, using the method of methylation-sensitive single nucleotide primer extension. In our study the (−820 to −601) region was examined and the methylation status of all 17 CpG sites was determined, with CpG-1 and CpG-2 being the sites inspected in Wolff et al. [7]. The Methyl-Profiler array is reporting on the average of detectable CpG sites over a region of 3,478 bp in the RUNX3 promoter (including the −820 to −601 region) and it relies on the presence of cleavage sites for methylation-sensitive and methylation-dependent restriction endonucleases for the detection. We and Kim et al. [22] inspected different regions of the RUNX3 gene and it is not unexpected that different patterns of methylation may be observed in normal urothelial cells. Likewise, the Methyl-Profiler may also be reporting on CpG sites that are outside of our region of inspection, giving a hypermethylated pattern. The actual sites reported on by the assay are proprietary information and not available to us. Our results on the methylation status of the RUNX3 CpG-1 and CpG-2 in the untreated control UROtsa cells, on the other hand, are fairly consistent with that reported by Wolff et al. [7]. We observed an average of 31% methylation in this cell line for these two CpG sites, whereas in seven matched normal/tumor cases reported in Wolff et al. [7], the average levels of methylation at these two sites were between 3−24% in the normal tissues but between 45−75% in the tumor tissues. For all 17 sites inspected in our study, only CpG-5 showed >60% methylation in the untreated UROtsa cells, but all other sites have <40% methylation, with 12 of the 17 sites at below 20% methylation. We consider the UROtsa cell line an appropriate model of normal urothelial cells for studying RUNX3 promoter methylation changes impacted by CSE. But even with the limitation of reporting false negatives and results that may appear inconsistent with previously published data for known loci, the Methyl-Profiler identified the IGF2 locus as presenting a hypomethylated phenotype in the chronically CSE-treated cells, a phenotype that was confirmed in the ensuing BS DNA sequencing of the IGF2 DMR.

Whereas hypermethylation in the RUNX3 tumor suppressor gene promoter is associated with bladder cancers of smokers [7], our chronic CSE treatment of cultured urothelial cells induced a hypomethylated phenotype at this locus over 4 of 17 CpG sites, with a significant increase in the fraction of the completely unmethylated haplotypes. So not only was CSE treatment unable to transform the urothelial cells the epigenetic changes in the RUNX3 tumor suppressor gene were of the opposite kind. One possible explanation is that the hypomethylation effect of CSE on RUNX3 in the cultured urothelial cells is the direct effect of CSE; but the hypermethylation of RUNX3 in bladder cancers of smokers is caused by additional indirect mediators, such as inflammatory cytokines. Indeed, serum concentrations of IL-6 were shown to be significantly increased in rats chronically treated with CSE [23]; while IL-6 could induce the expression of DNMT1 [24]. The actions of the inflammatory cytokines, either produced innately by the epithelial cells or perhaps more predominantly by the immune cells, may also be the necessary factors needed for cellular transformation. For example, IL-6 enhances urothelial cellular transformation in vitro [25]. Transformed cells have been shown to have concurrent up-regulation of DNA methyltransferases [26], so at the stage of fully transformed bladder cancer cells the RUNX3 promoter may after all manifest the hypermethylated phenotype in smokers. Our transient CSE treatment regimen (3 cycles but only 1 day) did result in an increase of IL-6 expression but the chronically CSE-treated cells did not show any CSE-associated IL-6 up-regulation other than what could be associated with passaging (***Figure 4C***). Previous studies showing increased IL-6 expression as a result of chronic CSE treatment involved constant exposure to this complex agent [23] but in our study we performed intermittent treatments with no CSE presence when the chronically treated cells were cultured between daily regimens. In our transient treatments the predicted IL-6 up-regulation phenotype was observed and we believe that the CSE treatment itself was appropriately performed. We at present do not have a testable hypothesis as to why the intermittent chronic treatment of UROtsa cells failed to up-regulate IL-6 relatively to the passage-matched untreated cells. We are performing experiments to determine if chronically treating the UROtsa cells with exogenous IL-6 or other inflammatory cytokines might be sufficient to transform the cells and/or induce hypermethylation.

CSE’s hypomethylation effect in the urothelial cells appears to be gene-specific, as the IGF2-H19 locus also showed a significantly reduced level of methylation in the CSE-treated cells along with RUNX3 but methylation at the PRSS8 locus or 16 other loci evaluated on the Methyl-Profiler array was not affected by the chronic CSE treatment. The hypomethylation of the RUNX3 and the IGF2-H19 loci may be characteristic of the responses of urothelial cells to CSE exposure without cellular transformation, which apparently requires more than just the CSE treatment. In the chronically CSE-treated UROtsa cells, the expression of the maintenance methylation enzyme, DNMT1, did not show CSE-associated changes (***Figure 5A***) but the expression of DHFR was significantly reduced at both the mRNA and the protein levels (***Figures 5B and 5C***). The reduced DHFR expression would result in a reduced supply of tetrahydrofolate, and in turn a reduced supply of methyl donor and a reduced rate of maintenance methylation, whereas upon continued DNA replication, the fractions of completely unmethylated haplotypes would then increase. This may account for the phenotype observed in our chronically CSE-treated cells. Alternatively, the CSE-associated hypomethylation phenotypes may be a result of higher activities of the double-stranded DNA break repair (DSBR) system at play. CSE is known to cause double-stranded DNA breaks when applied to human cells in culture [27]. DSBR has recently been shown to play a role in epigenetic reprogramming, producing completely unmethylated haplotypes in cultured cells upon introduction of DSBs [28]. CSE’s effect on DNA strand breaks is reversible in 24 hours upon removal of the CSE [29], allowing the cells to have at least the opportunity to recover from the damage and survive. In our case, our repetitive daily regimens of CSE treatment are expected to repeatedly produce the strand break and repair cycle, enabling the DSBR-mediated epigenetic reprogramming to generate the completely unmethylated haplotypes in the chronically CSE-treated cells at a much greater proportion (>20%, ***Figure 1B***) than those naturally occurring in the untreated control cells (< 2%, ***Figure 1B***). But clearly, both RUNX3 and PRSS8 were found to be hypermethylated in bladder cancers or bladder cancer cell lines, with RUNX3 hypermethylation directly associated with bladder cancers of smokers. With DSBR as a potential molecular mechanism to produce the hypomethylation phenotype, there is no direct conflict between the early-phase hypomethylation of select genes in CSE-treated urothelial cells and the hypermethylation of the same genes in the bladder cancer cells. Smokers are essentially playing the Russian Roulette with their urothelial cells by having the cyclic peaks of systemic CSE, which is expected to cause the DSBs and to activate the DSBR. In the early phase, the DSBR will produce the hypomethylation effect observed in our study in the marker genes evaluated. Eventually, some of the cells will not fully repair the DNA damage and will be transformed. The chromosomal DNA will then be at the mercy of the DNA methyltransferases which are expected to be up-regulated in the transformed cells, and the hypermethylation phenotype observed in bladder cancers may thus be manifested.

An alternative candidate mechanism for the CSE-induced hypomethylation phenotype is the involvement of enzymes that participate in active demethylation via various pathways such as oxidative demethylation, deamination of 5-methylcytosine to thymine followed by base excision repair, and nucleotide excision repair [30]. To test this hypothesis, we evaluated the effects of CSE on the expression of the TET (ten-eleven translocation) family proteins (TET1, TET2, and TET3), AID (activation-induced deaminase), the APOBEC (apolipoprotein B mRNA editing enzyme catalytic polypeptide) family protein APOBEC3a, and GADD45a (growth arrest and DNA-damage-inducible 45α). When the chronically CSE-treated UROCSE cells were compared with passage-matched untreated control UROtsa cells, none of the genes tested above showed statistically significant differential expression (data not shown). Thus it is unlikely that direct active demethylation could serve as a mechanism for the CSE-induced hypomethylation in the urothelial cells.

An immediately testable hypothesis for a molecular mechanism of the CSE-induced DHFR down regulation is that this effect is mediated by free radicals such as hydrogen peroxide, which has been known to be produced when cigarette smoke is bubbled through phosphate-buffered saline [31]; whereas DHFR down-regulation by hydrogen peroxide had previously been reported as a major molecular pathway regulating the DHFR gene expression [32]. We performed western blot analysis on UROtsa cells that were untreated, treated with CSE (by the intermittent regimen), treated with a free radical scavenger (catalase or glutathione), and treated with CSE but pretreated and treated along with the free radical scavengers. We were unable to show a rescue of the CSE-induced DHFR down-regulation with the free radical scavengers, while catalase rescued DHFR expression down-regulation induced by hydrogen peroxide treatment (data not shown), as previously described by others for another cell type [32]. The hydrogen peroxide produced by bubbling cigarette smoke through culture medium does not appear to be a major factor to cause the DHFR down-regulation. Alternative mechanisms of the CSE-induced DHFR down-regulation will be sought after in our future studies.

When we began this study our working hypothesis was that the RUNX3 tumor suppressor gene promoter is hypermethylated in bladder cancers of smokers so chronically treating normal urothelial cells with CSE might also lead to RUNX3 promoter hypermethylation, which is expected to down-regulated its transcription [33]. In the chronically CSE-treated urothelial cells, however, we observed a hypomethylation rather than a hypermethylation phenotype at the RUNX3 locus, with no apparent changes of transcription (***Figure 4A***). The UROtsa cells present a relatively low level of methylation at the RUNX3 gene, as stated above. Whereas a hypermethylation change could result in a down-regulation of RUNX3 expression [33], the hypomethylation change induced by the chronic CSE treatment is not expected to produce such a phenotype. On the other hand, the baseline methylation at RUNX3 in this cell line could have permitted the normal levels of expression thus the hypomethylation, most significantly in the form of the unmethylated haplotype would not be a sufficient change to up-regulate its expression, either. But the transient CSE treatment (1 day) clearly reduced RUNX3 transcription (***Figure 4A***). The molecular mechanism of this transcription down-regulation induced by the transient CSE treatment is unclear and will require future studies to uncover, but we can tentatively rule out a mechanism related to gross changes in DNA methylation.

Both IGF2 and H19 were found to be up-regulated in the airway epithelial cells after chronic CSE treatment from the mono-allelically expressed chromosome with no evidence for loss of imprinting [8]. In the chronically CSE-treated urothelial cells however, H19 was down-regulated (***Figure 4B***). The IGF2-H19 locus is imprinted in normal human bladder epithelial cells [34], but the data from our untreated control UROtsa cells indicate that the imprinting is lost in this cell line. The UROtsa was immortalized by SV40-T and the loss of imprinting at the IGF2-H19 locus could be caused by SV40-T expression which has been shown to induce DNA hypermethylation [35]. The loss of imprinting in the parental UROtsa cells however, does not take away the validity of the hypomethylation effect of CSE exposure in the early phases as the non-imprinted RUNX3 also showed this phenotype.

On the practical application side, one of our intents of the present study was to identify tumor suppressor gene loci that show early epigenetic changes in urothelial cells exposed to CSE for use as potential diagnostic markers. Urothelial cells directly exposed to CSE, simulating exposure to the systemic soluble contents of cigarette smoke, have a hypomethylated phenotype in genes that show hypermethylation in bladder cancers. Efforts directed at developing epigenetic tests for early detection and diagnosis of bladder cancer should take this phenomenon under careful consideration.

## Supporting Information

Figure S1 **Methyl-Profiler PCR Array analysis of tumor suppressor gene methylation changes.** Genomic DNA extracted from UROtsa cells chronically treated with CSE, UROCSE-p32 (32^nd^ passage) and from untreated control cells, UROtsa-p32 (32^nd^ passage), were applied to the arrays according to the supplier’s procedures. HM: Hyper-methylated; IM: Intermediately-methylated; UM: Un-methylated per supplier’s criteria. Failure: reactions failed to pass the supplier’s quality control standards.
(TIF)
