# Supplementary figures and images for: Long-Term Exposure to Cigarette Smoke Extract Induces Hypomethylation at the RUNX3 and IGF2-H19 Loci in Immortalized Human Urothelial Cells
Source: PLoS One. 2013 May 28;8(5):e65513. doi: 10.1371/journal.pone.0065513 (PMC3665628; doi:10.1371/journal.pone.0065513)

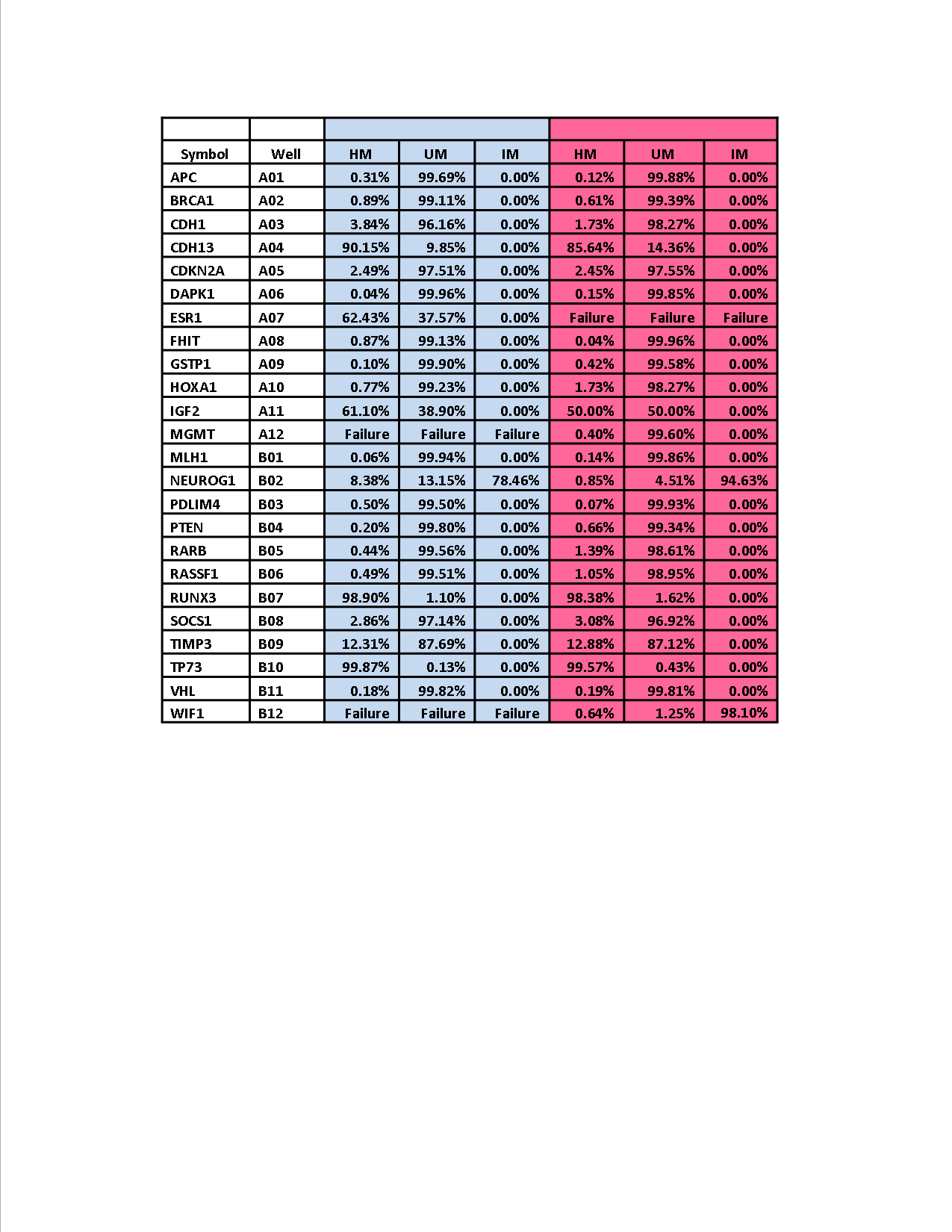

Supplement: Figure S1 — Methyl-Profiler PCR Array analysis of tumor suppressor gene methylation changes. Genomic DNA extracted from UROtsa cells chronically treated with CSE, UROCSE-p32 (32nd passage) and from untreated control cells, UROtsa-p32 (32nd passage), were applied to the arrays according to the supplier’s procedures. HM: Hyper-methylated; IM: Intermediately-methylated; UM: Un-methylated per supplier’s criteria. Failure: reactions failed to pass the supplier’s quality control standards. (TIF) [file pone.0065513.s001.tif]
